# Vitamin D supplementation reduced blood inflammatory cytokines expression and improved graft function in kidney transplant recipients

**DOI:** 10.3389/fimmu.2023.1152295

**Published:** 2023-07-06

**Authors:** Yang-Juan Bai, Ya-Mei Li, Shu-Meng Hu, Yuan-Gao Zou, Yun-Fei An, Lan-Lan Wang, Yun-Ying Shi

**Affiliations:** ^1^ Department of Laboratory Medicine/Research Centre of Clinical Laboratory Medicine, West China Hospital, Sichuan University, Chengdu, China; ^2^ Department of Nephrology, West China Hospital, Sichuan University, Chengdu, China

**Keywords:** kidney transplantation, vitamin D, chronic allograft dysfunction, inflammation, cytokine

## Abstract

**Background:**

Chronic allograft dysfunction(CAD) is the leading cause of graft loss in kidney transplant recipients (KTRs). Inflammatory process is believed to be one of the major contributors to CAD. The aim of this study is to explore the anti-inflammatory effect of vitamin D (VD) supplementation in KTRs and its role in the graft function improvement(protection).

**Methods:**

A retrospective cohort of 39 KTRs with chronic antibody mediated rejection(CAMR)or stable renal function and a prospective cohort of 42 KTRs treated or untreated with VD were enrolled. Serum levels of vitamin D metabolism and serum inflammatory cytokines, renal graft function, and routine blood biomarkers were tested and dynamically tracked within 12 months post-transplant.

**Results:**

Compared with the stable group, the CAMR group exhibited significantly elevated serum levels of inflammatory cytokines IL-1β, IFN-γ, IL-2, IL-10, IP-10, and HMGB1 (P <0.05). The supplementation of vitamin D effectively increased the serum concentration of vitamin D in kidney transplant recipients (KTRs) in the treated group. During the course of treatment, the treated group exhibited a gradual increase in eGFR levels, which were significantly higher than those observed in the untreated group at 12 months post-transplant (p<0.05). Notably, as eGFR improved, there was a significant decrease in levels of IL-1β, IFN-γ, IL-2, IL-10, IP-10 and HMGB1 in the treated group compared to the untreated group (P<0.05).

**Conclusion:**

This study confirmed that immune-inflammation is a crucial factor in the development of CAD in KTRs.VD deficiency impairs its anti-inflammatory activity. By assisting in the regulation of excessive immune inflammation and restoration of immune homeostasis, effective VD supplementation contributes to protection and maintenance of graft function in KTRs.

## 1 Introduction

Kidney transplantation is considered the optimal renal replacement therapy (RRT) for patients diagnosed with end-stage renal disease (ESRD). With the advancement of surgical techniques and immunosuppressive medications, the incidence of acute rejection following kidney transplantation (KT) has significantly decreased, and the short term outcomes of KT has been significantly improved. However, graft function maintenance and the long-term survival of kidney transplant recipients (KTRs) remain challenging in post-transplant management.

Most of the studies showed a negative effect of vitamin D deficiency on kidney transplantation by being associated with a worse graft function, higher incidence of acute rejection episodes, higher incidence of proteinuria and lower overall graft and patient survival rate (1).It is well-established that Vitamin D (VD) plays a crucial role in mineral and bone metabolism as well as the treatment of secondary hyperparathyroidism in patients with chronic kidney diseases (CKD). Recently, there has been an increasing focus on the immunomodulatory effects of VD in KTRs.

Vitamin D is a fat-soluble vitamin, with ergocalciferol (vitamin D2, VD2) and cholecalciferol (vitamin D3, VD3) being the two primary native forms of this nutrient. The former is derived from fungi and plants, while the later is synthesized from 7-dehydrocholesterol in the epidermal layer of the skin under the UVB irradiance or absorbed via the intestine from animal-based foods. Both compounds are modified in 25-hydroxyvitamin D (25(OH)D) by 25-hydroxylase in the liver, and then 25(OH)D is hydroxylated in 1,25-dihydroxyvitamin D [1,25(OH)2D] by 1α-hydroxylase (CYP27B1). 1,25-(OH)2-VD, as the main active metabolite of VD, can bind to and activate the vitamin D receptor (VDR). The activated VDR binds to the retinoid X receptor (RXR) and works as a heterodimer to the VD response elements of related genes (e.g. calcium-binding protein gene, osteocalcin gene, etc.) to regulate the expression of related genes and play bioregulatory roles (2).

Growing evidences show that VD is not only involved in calcium, phosphorus and bone metabolism, but also an important immunomodulatory molecule. Based on numerous studies in animal models and human, a substantial role of VD has been found to be involved in the maintenance of immune homeostasis (2, 3).Insufficiency of VD is associated with abnormal immune responses. A series of studies reported that reduced VD levels act as a driver of systemic chronic inflammation (4), and is associated with an increased risk of various cancers (5), progression of infectious diseases and inflammatory autoimmune diseases (6). Several *in vivo* and *in vitro* studies have confirmed that VD plays an anti-inflammatory role in a variety of diseases (2).

The outcome of the graft is determined by the delicate balance between pro-inflammatory and anti-inflammatory effects of the immune system. The cytokine storm generated by the immune response after transplantation can lead to rejection, which interferes with the graft function and survival. It has been reported inflammation in the early phase after kidney transplantation was associated with increased long-term all-cause mortality (7).In our previous research, we discovered a close correlation between chronic renal graft failure and the persistent inflammatory response triggered by an imbalance in cellular Th17/Treg and Tfh/Tfr ratios among KTRs (8, 9). However, there is a paucity of relevant studies investigating the association between peripheral blood VD levels and expression of inflammatory cytokines in KTRs with chronic antibody mediated rejection(CAMR). Furthermore, prospective studies examining the potential role of VD supplementation in modulating immune inflammation following transplantation are scarce.

The objective of this investigation was to examine the metabolic status of vitamin D in kidney transplant recipients with chronic rejection and investigate the association between alterations in vitamin D levels and immuno-inflammatory cytokines by analyzing differences in peripheral blood vitamin D levels and inflammatory cytokines between kidney transplant recipients with chronic rejection and those with stable graft function. Additionally, a prospective dynamic research was conducted on renal function, VD levels, and the expression of inflammatory cytokines in a cohort of kidney transplant recipients receiving VD supplementation to investigate the targets of action for VD supplementation on immune-inflammatory status and graft kidney function maintenance.

## 2 Materials and methods

### 2.1 Description of the cohort

#### 2.1.1 Retrospective cohort 1: KTRs with chronic antibody mediated rejection or stable renal function

Nineteen KTRs with biopsy-proven chronic antibody mediated rejection (CAMR) and twenty matched (posttransplant time, age, and gender) KTRs with stable renal function (eGFR>60 ml/min/1.73m^2^ at least three months before enrollment) were enrolled, referred as CAMR group and stable group respectively. All the recipients attended at West China Hospital of Sichuan University from January 2020 to December 2020. All enrolled KTRs received triple maintenance immunosuppressive therapy consisting of tacrolimus (Tac), mycophenolate mofetil (MMF), and corticosteroids. None of the enrolled KTRs received VD supplementation after transplantation. Exclusion criteria: 1) ABO blood type incompatible between donor and recipient; 2) KTRs with chronic infectious diseases such as tuberculosis and hepatitis; 3) KTRs with neoplastic diseases; 4) KTRs with autoimmune diseases.

#### 2.1.2 Prospective cohort 2: KTRs with or without VD supplementation

Cohort 2 consisted of 20 KTRs receiving VD supplementation (referred as the treated group) and 22 KTRs without VD supplementation (referred as the untreated group), which was randomly drawn from our group’s prospective clinical trail study of VD supplementation for KTRs with secondary hyperparathyroidism (Chi CTR2200056077) (10). In Patients who underwent allogeneic KT at West China Hospital of Sichuan University between June 2020 and December 2020 were selected and screened according to the inclusion and exclusion criteria. Informed consent were obtained after surgery. All enrolled KTRs received basiliximab intravenously as induction immunosuppressive therapy. Methylprednisolone at 500 mg/day was administrated intravenously on the operation day and the following 2 days after transplantation. Prednisolone was then administered orally and tapered rapidly from 1 mg/kg/day to 5–10 mg/day within 7 days for long-term maintenance. Standard triple maintenance immunosuppressive therapy consisting of tacrolimus (Tac), mycophenolate mofetil (MMF), and corticosteroids was administered to the KTRs. The dosage of Tac and MMF were adjusted to reach a stable plasma concentration between 2 and 4 weeks after transplantation. Inclusion criteria: 1) 18-65 years old; 2) first allogeneic kidney transplantation; 3) PTH ≥ 65 pg/mL within 1 month after kidney transplantation.4) a maintenance immunosuppressive regimen of Tac, MMF and prednisone. Exclusion criteria: 1) delayed graft function (DGF) after kidney transplant; 2) bleeding exploration of the grafted kidney or removal of the grafted kidney after transplantation; 3) parathyroidectomy prior to kidney transplant; 4) ABO blood type incompatible between donor and recipient; 5) KTRs with chronic infectious diseases such as tuberculosis and hepatitis; 6) KTRs with neoplastic diseases; 7) KTRs with autoimmune diseases; 8) history of primary parathyroid disease or other disorders of calcium and phosphorus metabolism prior to KT; 9) patients who cannot be followed up in our hospital after KT. Since not every physician held the same opinion on the necessity of early treatment of hyperparathyroidism, whether the KTRs included received treatment for VD supplementation was dependent on the patients’ and physicians’ own will. The posttransplant VD supplementation regimen was based on the patients’ PTH, 25(OH)VD and serum Ca levels. For KTRs with VD insufficiency (serum 25(OH)VD <30 ng/mL), a starting dose of 2000 IU cholecalciferol daily was given and serum 25(OH)VD levels were monitored every 3 months posttransplant. For KTRs whose VD insufficiency has been corrected, the cholecalciferol dose would be reduced to 400-800 IU daily to maintain a normal VD level.

This study was approved by the Ethics Committee of West China Hospital, and written informed consent was obtained from each participant before enrolment. The study was preregistered in the Chinese Clinical Trial Registry, and the register number is ChiCTR2200056077.

### 2.2 Sample and data collection

Baseline demographic characteristics, clinical and laboratory tests data were collected for all enrolled participants. All kidney transplant recipients (KTRs) received regular follow-up care, with immunosuppressive drug regimens strictly adjusted based on therapeutic drug monitoring by physicians at West China Hospital.

For the cohort of KTRs with chronic rejection or stable renal function, fresh fasting serum samples were collected at their outpatient visits and stored at -80C° for the determination levels of VD and inflammatory cytokines.

For the cohort of KTRs with or without VD supplementation, patients were followed up regularly at our hospital. Fresh fasting serum samples were collected at 2 weeks, 3, 6, 12 and 18 months posttransplant and frozen at -80°C for the determination levels of VD and inflammatory cytokines.

### 2.3 Serum vitamin D level determination

Serum 25(OH)VD3 and 25(OH)VD2 levels were determined using ultra performance liquid chromatography-tandem mass spectrometry (UPLC-MSMS) and the sum of the two was calculated as the serum 25(OH)VD concentration. We used mass spectrometry assay kit for Vitamin D Metabolites (Pinsheng, Fuzhou, China) and a triple quadrupole LC–MS/MS consisted of a ACQUITY UPLC system in combination with a Xevo TQ-S triple quadrupole mass spectrometer, from Waters(Milford, USA) to perform the 25(OH)VD concentration assay.

### 2.4 Serum inflammatory cytokines determination

Serum inflammatory cytokines IL-1β, IL-2, IL-4, IL-10, IL-12, IFN-γ, IP-10 and HMGB1 were determined by using the Millipore^®^ HSTCMAG-28SK kit and Bio-Plex^®^ 200 Suspension Chip System.

### 2.5 Statistical analysis

Normality tests of continuous variables were performed with the Shapiro–Wilk test. Variables with a normal distribution are displayed as the mean ± s.d. and were compared using independent samples t-test or ANOVA. Asymmetric variables were reported as the median (interquartile range), and comparisons were assessed with the Mann–Whitney U test and Kruskal–Wallis test. Categorical variables were compared using the chi-square test or Fisher’s exact test. A two-tailed *P <*0.05 was considered statistically significant. All statistical analyses were performed using Empower^®^ (www.empowerstats.com, X&Y Solutions Inc., Boston MA) and SPSS version 28.0 statistical software (SPSS Company).

## 3 Results

### 3.1 Description of the study population

A total of 39 KTRs were enrolled in the retrospective cohort (19 recipients in CAMR group and 20 recipients in stable group) and the characteristics were shown in **Table 1**. There was no significant difference in general demographic information, pretransplant history, and basic clinical background between the two groups. The Tac concentration was within 4.2-6.8ng/mL, and the area under the curve of mycophenolate acid (MPA) was controlled within 57.9–78.6mg·h/L.

**Table 1:** Characteristics of allo-renal recipients in chronic rejection group and stable group.

	CAMR group (n=19)	Stable group (n=20)
Age	38.0 ± 5.6	40.2 ± 7.2
Gender (M/F)	15/4	15/5
HLA mismatch	4 (4-6)	4 (3-5)
Time after Tx, years	3.0 (1.0-5.0)	4.5 (3.0-6.5)
Donor type		
DCD	4 (21.05%)	4 (20.00%)
Living relatives donation	15 (78.95%)	16 (80.00%)
Dialysis duration,year	2 (1-3)	2 (1-4)
TAC-C0, ng/ml	5.6 (4.3-6.8)	5.0 (4.2-6.0)
MPA AUC,mg·h/L	68.30 ± 10.32	63.40 ± 3.48
Renal function		
SCR, μmol/L	171.45 ± 121.87*	100.34 ± 20.16
eGFR,ml/min/1.73m^2^	46.77 ± 14.12*	76.98 ± 15.08
Liver function		
ALT, IU/L	18 (16-27)	14 (10-22)
AST, IU/L	19 (17-23)	17 (15-21)

* p<0.05 compared with stable group

A total of 39 KTRs were enrolled in the retrospective cohort study, with 19 patients in the CAMR group and 20 patients in the stable group. The characteristics of these patients are presented in **Table 1**. No significant differences were observed between the two groups regarding general demographic information, pretransplant history, or basic clinical background. Tacrolimus concentration was maintained within a range of 4.2-6.8 ng/mL, while mycophenolic acid (MPA) exposure was controlled at an AUC(area under the curve)of 57.9-78.6 mg·h/L.

A total of 42 KTRs were included in a prospective cohort of VD supplement treatment (20 in treated group and 22 in untreated group) and the characteristics are shown in **Table 2**. Tacrolimus concentration was maintained within a range of 5.95–7.11 ng/mL, while mycophenolic acid (MPA) exposure was controlled at an AUC(area under the curve)of 45.7–70.9 mg·h/L. The treated group received VD supplementation starting at 2 (1-4) weeks after transplantation.

**Table 2:** Characteristics of allo-renal recipients in the VD supplementation treatment cohort.

	Treated group (n=20)	Untreated group (n=22)
Age	37.1 ± 12.2	38.1 ± 12.0
Gender (M/F)	11/9	13/9
HLA mismatch	4 (4-6)	4 (3-5)
Donor type		
DCD	3 (15.00%)	3 (13.64%)
Living relatives donation	17 (85.00%)	19 (86.36%)
Dialysis duration,year	2 (1-3)	2 (1-4)
Postoperative anti-THPT therapy regimen		
Vitamin D	11 (55.00%)	None
calcitriol	3 (15.00%)	None
calcitriol+Vitamin D	5 (15.00%)	None
Sinakase+Vitamin D	1 (5.00%)	None
TAC-C0, ng/ml		
2 weeks	5.86 ± 1.23	6.03 ± 1.17
3 months	6.83 ± 1.33	7.12 ± 1.19
6months	6.52 ± 1.26	6.80 ± 1.25
12months	6.37 ± 1.11	6.51 ± 1.27
18months	6.3 ± 1.24	6.78 ± 1.36
MPA AUC,mg·h/L		
2 weeks	67.30 ± 9.42	65.30 ± 8.48
3 months	63.20 ± 9.36	61.90 ± 7.31
6months	60.50 ± 7.32	63.10 ± 9.51
12months	58.30 ± 11.32	60.40 ± 7.48
18months	60.80 ± 8.38	61.20 ± 8.57
Liver function		
2 weeks ALT, IU/L	18 (16-27)	14 (10-22)
2 weeks AST, IU/L	19 (17-23)	17 (15-21)
3 months ALT, IU/L	17 (15-26)	15 (9-23)
3 months AST, IU/L	20 (16-23)	18 (16-22)
6 monthsALT, IU/L	18 (15-26)	15 (11-21)
6 months AST, IU/L	19 (16-24)	17 (13-20)
12 months ALT, IU/L	18 (15-26)	15 (12-20)
12 months AST, IU/L	16 (14-21)	16 (14-20)
18 months ALT, IU/L	19 (15-24)	17 (12-22)
18 months AST, IU/L	16 (14-20)	17 (15-20)

### 3.2 Serum VD, inflammatory cytokines analysis in KTRs with chronic rejection or stable renal function

#### 3.2.1 The serum levels of vitamin D were found to be deficient in KTRs

**Figure 1:**
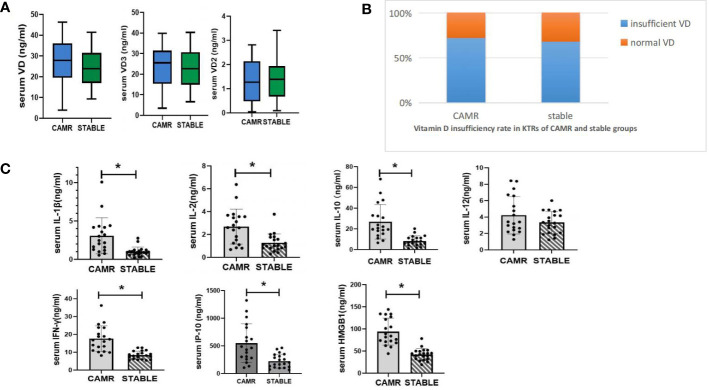
Serum vitamin D metabolism status and serum inflammatory cytokine concentration in CAMR and stable kidney transplant recipients. **(A)** serum vitamin D concentration of CAMR and stable kidney transplant recepients (ng/ml). **(B)** Vitamin D insuffieciency rate in LTRs of CAMR and stable groups. Vitamin D insufficiency was define as serum vitamin D < 30 ng/ml. **(C)** serum concentrations of inflammatory cytokines in CAMR and stable kidney transplant recepients; *p<0.05, vs stable group.

The average serum 25(OH)VD concentrations of KTRs in the CAMR group and the stable group were both lower than 30ng/ml, and there was no significant difference in serum 25(OH)VD levels between the two groups (*P >*0.05).The VD insufficiency rates were 72.4% and 68.3% respectively in CAMR and stable groups. The results were shown in **Figure 1**.

#### 3.2.2 Serum inflammatory cytokines levels were elevated in CAMR group

**Figure 2:**
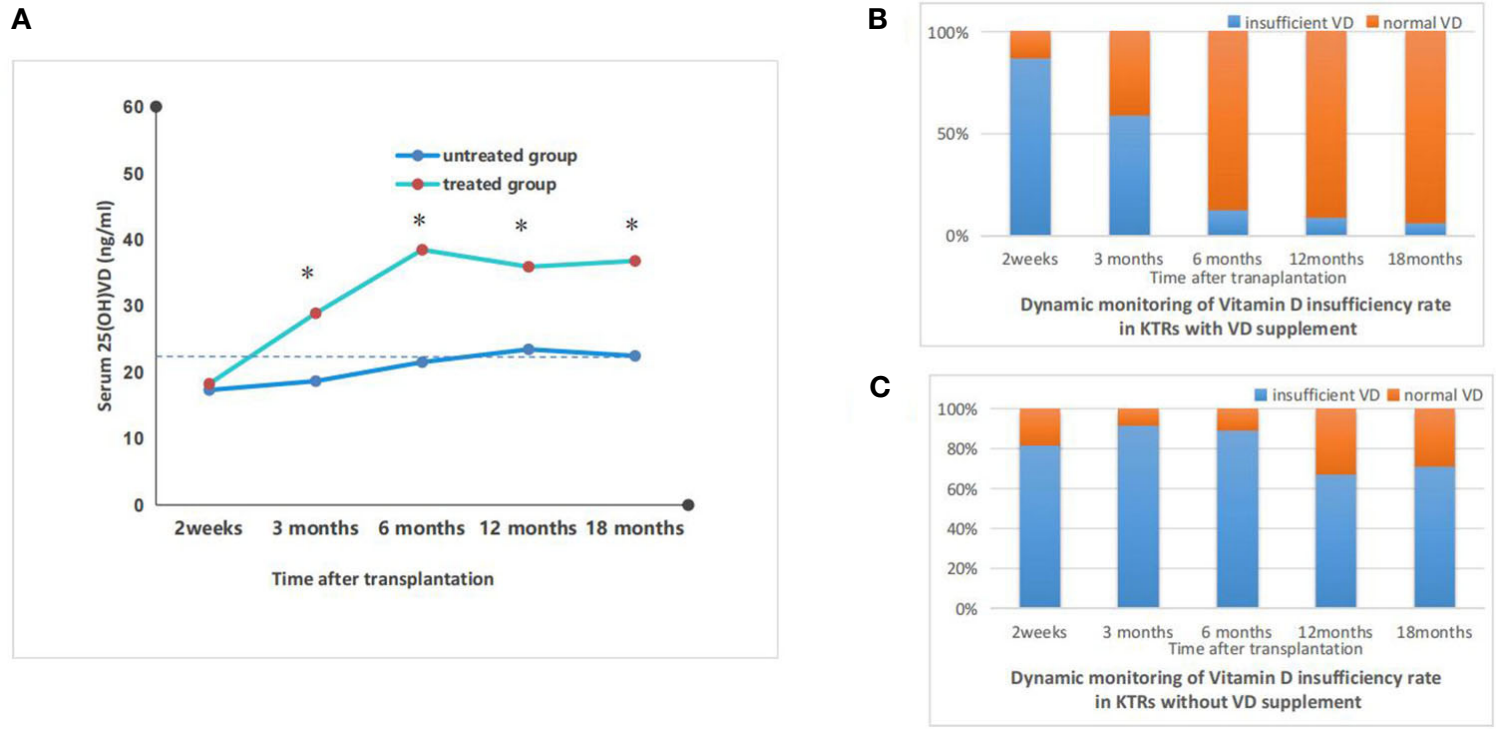
Dynamic monitoring of vitamin D metabolism status in vitamin D treated vs untreated kidney transplant recipients. **(A)** the dynamics monitoring of average serum vitamin D concentraions of kidney transplant recipients with or without vitamin D supplement; *p<0.05, treated vs untreated group; **(B)** the dynamic monitoring of vitamin D insufficiency rate in KTRs with vitamin D supplement. Vitamin D insufficiency was defined as serum vitamin D < 30ng/ml; **(C)** the dynamic monitoring of vitamin D insufficiency rate in KTRS withour vitamin D supplement. Vitamin D insufficiency was defined as serum vitamin D < 30ng/ml.

Analysis of serum inflammatory cytokines in KTRs showed that serum IL-1β, IFN-γ, IL-2, IL-10, IP-10 and HMGB1 levels in CAMR group were significantly higher than those in stable group (*P <*0.05), and serum IL-12 level was similar in these two groups (*P >*0.05).The results were shown in **Figure 2**.

### 3.3 Dynamic monitoring of serum VD levels, renal function and inflammatory cytokines in KTRs receiving vitamin D supplement treatment

#### 3.3.1 The impact of vitamin D supplementation on serum levels of VD

**Figure 3:**
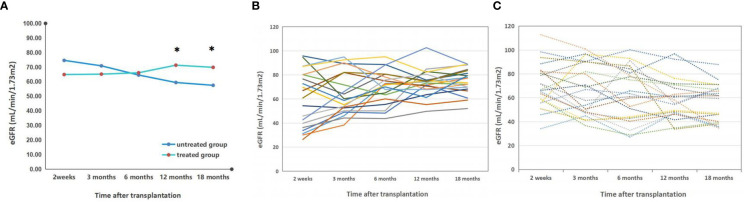
Dynamic monitoring of eGFR in vitamin D treated vs untreated kidney transplant recipients. **(A)** the dynamic monitoring of average eGFR in kidney transplant recepients with or without vitamin D supplement; *p<0.05, treated vs untreated group; **(B)** the individual dynamic monitoring of eGFR in kidney transplant recepient rate with vitamin D supplement (treated group); **(C)** the individual dynamic monitoring of eGFR in kidney transplant recepient rate without vitamin D supplement (untreated group).

In treated group, serum 25(OH)VD levels significantly increased and the proportion of patients with VD deficiency dramatically decreased. The treated group exhibited significantly higher serum levels of 25(OH)D compared to the untreated group, starting from 3 months post-transplant. Additionally, the proportion of patients with VD deficiency was significantly lower in the treated group than in the untreated group. The serum levels of 25(OH)VD in treated group peaked at 6 months post-transplant and declined slightly thereafter and remained above 30ng/ml. The serum levels of 25(OH)VD in the untreated group increased slightly with time after transplantation, but still remained below 30 ng/ml and the VD deficiency rates remained above 65%. The results were shown in **Figure 3**.

#### 3.3.2 The impact of vitamin D supplementation on renal graft function

**Figure 4:**
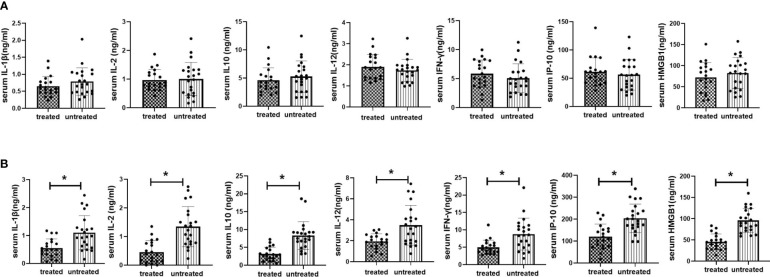
Serum inflammatory cytokine expression in vitamin D treated vs untreated kidney transplant recipients at 2 weeks and 12months after transplantation. **(A)** Serum inflammatory cytokine expression in vitamin D vs untreated kidney transplant recipients at 2 weeks after transplantation; **(B)** Serum inflammatory cytokine expression in vitamin D treated vs untreated kidney transplant recipients at 12 months after transplantation. *p<0.05, treated vs untreated group.

The eGFR levels in the treated group remained stable from 2 weeks to 6 months after transplantation and did not differ significantly from those in the untreated group. During the course of treatment, the eGFR levels in the treated group gradually increased and surpassed those in the untreated group at 6 months post-transplantation. At 12 months post-transplantation, there was a significant difference between the eGFR levels in the treated (71.34 ± 10.57) and untreated groups (59.47 ± 12.79), (p<0.05); this disparity persisted at 18 months post-transplantation. The results were shown in **Figure 4**.

#### 3.3.3 The impact of vitamin D supplementation on serum levels of inflammatory cytokines

To investigate the effect of VD supplementation on inflammatory status in KTRs, we analyzed multiple inflammatory cytokines in both treated and untreated groups at 2 weeks (starting point of VD supplementation) and 12 months (starting point of significant difference in eGFR between treated and treated groups) after kidney transplantation. No significant difference in inflammatory cytokine levels was found between treated and untreated groups at 2 weeks post-transplant. At 12 months post-transplant, the levels of IL-1β, IFN-γ, IL-2, IL-10, IP-10, and HMGB1 were significantly lower in treated group than those in untreated group (*P*<0.05). The results were shown in **Figure 4**.

## 4 Discussion

A diverse range of immune cells within the body, including CD4+ T cells, CD8+ T cells, monocytes, macrophages and dendritic cells express the vitamin D receptor (VDR). Vitamin D plays a multifaceted regulatory role in both innate and adaptive immune responses, with deficiency linked to aberrant immune function. VD deficiency is prevalent among KTRs due to multiple factors. Reduced VD levels in KTRs are linked to an increased risk of post-transplant infections, tumors, cardiovascular disease, disorders of mineral and bone metabolism, as well as post-transplant diabetes (11–14). It has been reported that recipients with sufficient levels of VD at 10 weeks post-transplant exhibit a higher survival rate compared to patients with VD deficiency or insufficiency (11). Studies had shown that low 25(OH)D levels in KTRs was independently associated with an increased risk of all-cause mortality (14). Available reports suggested a potential impact of VD on the maintenance of transplanted kidney function. The available reports indicate a potential association between VD and the preservation of renal function in KTRs. In KTRs, it has been reported that VD deficiency is accompanied by reduced eGFR, increased risk of graft rejection and infection (11). There was a trend towards increased eGFR (levels ≥20ng/mL) 1 year after transplantation in recipients without VD deficiency. *De-novo* donor-specific antibody (dnDSA) was associated with posttransplant VD deficiency, low VDR activity and reduced graft eGFR 12 months after transplantation (13).

Our previous research has focused on investigating the role of T lymphocytes and their sub-populations in the regulation of transplant immunity, and We observed that an imbalance in the Th17/Treg cell ratio was linked to graft failure in KTRs (8, 9). Th17/Treg imbalance has been shown to be strongly associated with chronic inflammatory damage in a variety of diseases, and studies from autoimmune diseases showed that correcting Th17/Treg balance shift and chronic inflammatory states is one of the priorities for improving treatment outcomes (15, 16). It has been demonstrated that active VD inhibits the development of Th17 effector cells, suppresses IL-17 production and simultaneously induces Treg proliferation, upregulates IL-10 expression and exerts anti-inflammatory effects (10, 17, 18). With further research and understanding of immune mechanisms, the immunomodulatory effects of VD on transplant rejection and its clinical therapeutic potential have become a focal point for researchers and clinicians. In this study, we focused on the regulatory effect of VD on the expressions of inflammatory cytokines as well as allo-graft function in KTRs. In addition to retrospective cohort, based on our prospective study investigating the role of VD supplementation in maintaining normal bone metabolism and preventing secondary hyperparathyroidism after renal transplantation (10), we monitored the dynamic changes of peripheral blood VD levels, inflammatory cytokine expressions and renal function in KTRs after VD supplementation.

Immunoinflammatory cytokines released by the immune cells upon stimulation to pathogen-associated pattern molecules (PAMP) or damage-associated pattern analysis (DAMP) signaling are important components of the inflammatory vesicles, and are key substances for initiating immune response. DAMP, which consists of endogenous molecules produced and released by damaged and dead cells *in vivo* in the transplantation immune response, is important in inducing transplantation rejection. IL-1β, IFN-γ, IL-2, IL-12, IL-10, IP-10, and HMGB1 are all inflammatory cytokines secreted by innate or adaptive immune cells upon activation, and these molecules play a crucial role in the regulation of alloimmunity and immune inflammation (19).IL-1β is an important pro-inflammatory factor, mainly expressed by natural immune cells such as monocytes and DCs. IL-1β can act directly on Th cells and assist in their activation and at the same time is also directly involved in local or systemic inflammatory responses. IFN-γ can be produced by NK cells, Th1, CD8 T cells and γδ T cells. It can induce and enhance the expression of MHC class I/II molecules in a variety of cells to enhance the immune response (promoting T cell differentiation, enhancing killing function and promoting the involvement of macrophages) and plays an important role in the maintenance of the chronic inflammatory response. IL-12 is an important link between intrinsic and adaptive immunity which mainly acts on T cells and NK cells, promotes the differentiation of CD4+T cells to Th1 and Tfh and the secretion of IL-2 and IFN-γ and enhances the killing activity of NK and CD8+T cells. IL-10 can be produced by DCs, macrophages, NK cells and other intrinsic immune cells. It is a negative cytokine that mediates immunosuppressive effects. HMGB1 is a ubiquitous nuclear protein which can be actively or passively released into the extracellular compartment due to cell necrosis or macrophage activation. As an important DAMP molecule, HMGB1 is exerts a powerful pro-inflammatory function by activating macrophages, inducing DC maturation and Th1 cell polarization and is a major mediator of late inflammation. HMGB1 can stimulate DC to secrete IL-6 and induce alloreactive CD4+T cells to differentiate into Th17 and recruit neutrophils to the local graft aggravating graft injury. The increased expression and release of HMGB1 is involved both in acute and chronic organ transplant rejection and graft tissue injury (20, 21).

In this study, our data revealed significantly elevated expressions of various inflammatory cytokines (IL-1β, INF-γ, IL-2, IP-10, HMGB1) in the peripheral blood of KTRs with chronic rejection compared to those with stable renal function. This suggests that multiple types of immune cells including monocytes, neutrophils, T cells, Th1 cells, NK cells involved in the immune inflammatory response were activated in KTRs with chronic rejection. IL-10, as a negative regulator, its high expression in the chronic rejection group is a sign of feedback suppression of inflammatory activity of immune cells. Single cell sequencing studies of PBMC in renal transplant recipients revealed (22) that most cell types in CAMR patients exhibit a strong interferon response and release of pro-inflammatory cytokines. A variety of genes involved in pro-inflammatory responses and immunomodulatory related genes (e.g. MTND6, CXCL8, NFKBIA, NFKBIZ) showed high expression in T and B cells. Combined with the results of this study, it suggests that the persistence of an active state of chronic inflammation and its associated tissue damage are important immune factors contributing to chronic graft loss in kidney transplant recipients. Therefore, controlling the activation of immune cells and inflammatory effector cells as well as reducing the release of inflammatory effector molecules should be considered as important therapeutic targets for preventing and managing chronic graft rejection and maintaining and protecting the function of transplanted organs.

It has been reported that VD deficiency is associated with a decrease in eGFR and an increase in the risk of graft rejection, infection, and cardiovascular events among kidney transplant recipients (8, 9). In this study, serum VD levels were below the lower limit of the normal reference range in both the chronic rejection group and stable group, indicating that VD insufficiency is prevalent among KTRs. Consistent with previous reports, there was no statistically significant difference in serum VD levels between the two groups of KTRs (11–14).Despite the multiple causes for the prevalence of VD insufficiency or deficiency in KTRs, there are no clear guidelines for routine VD supplementation after renal transplantation. At present, most clinicians and clinical studies focus on the relationship between insufficiency of VD and abnormal bone metabolism and secondary hyperthyroidism (14, 16), while few studies focus on the relationship between VD level and immune inflammatory cytokines which mediated chronic rejection. Our data demonstrated that, even up to 18 months post transplantation, renal transplant recipients who did not receive VD supplementation experienced only a slight increase in serum VD levels over time and remained in a state of VD insufficiency. Simultaneous dynamic monitoring of renal function revealed a gradual decline in eGFR levels among KTRs in the untreated group over time after renal transplantation, indicating an increased risk for graft dysfunction. In sharp contrast, KTRs receiving post-transplant VD supplementation showed a significant increase in serum VD levels and effective correction of VD deficiency (mean VD concentration was above the lower limit of the reference range, the VD deficiency rate was reduced from 87.04% to less than 10%) and the VD concentration reached its peak level 6 months after transplantation. Simultaneously, the treated group exhibited a concomitant recovery of renal function, as evidenced by a gradual increase in eGFR levels following VD supplementation. Notably, both serum VD and eGFR levels in KTRs of the treated group demonstrated a break point at 6 months post-transplantation. Subsequently, VD levels remained adequate while eGFR continued to rise and was significantly higher than that observed in the untreated group from 12 months post-transplant on (p<0.05). The findings suggest a close association between effective vitamin D supplementation and the preservation and enhancement of renal function in KTRs.

*In vitro* studies have demonstrated that the active form of vitamin D can modulate various immune cells by regulating cytokine and chemokine expression and secretion, leading to inhibition of IL-2, IFN-γ, TNF-α, IL-17, and IL-21 production while promoting anti-inflammatory cytokine IL-10 synthesis, ultimately resulting in suppression of proinflammatory states (17, 23–26). However, the reports on the anti-inflammatory effects of vitamin D in patient treatment and prevention of related diseases are limited and controversial (6, 26, 27), especially in the kidney transplant recipients. Paricalcitol treatment was observed to significantly decrease serum levels of IL-6, TNF, and TGF-βin kidney transplant recipients with secondary hyperparathyroidism or proteinuria. However, it was also noted that circulating high sensitivity CRP and a range of cytokines and inflammatory molecules were not significantly affected by paricalcitol treatment in this population (28–31). The network of inflammatory cytokines and molecules is intricate. Our retrospective cohort data has demonstrated the involvement of a series of inflammatory cytokines in the immune-inflammatory response among KTRs with chronic rejection, which indicates that anti-inflammatory therapy may confer significant protective effects on the transplanted kidney.

To investigate the potential anti-inflammatory effects of vitamin D (VD) supplementation and its role in protecting graft function, we monitored changes in serum levels of inflammatory cytokines between treated and untreated groups at 2 weeks and 12 months post-transplantation. At the beginning of VD supplementation (2 weeks post-transplantation), there were no significant differences in serum levels of IL-1β, INF-γ, IL-2, IP-10, HMGB1, IL-12, and IL-10 between the two groups. At 12 months after transplantation, when there was a significant difference in eGFR between the treated and untreated groups, significant differences were found in the peripheral inflammatory cytokines expressions between these two groups: the serum levels of IL-1β, INF-γ, IL-2, IP-10, HMGB1, IL-12, and IL-10 were significantly lower in the treated group than those in the untreated group (p<0.05). At 12 months post-transplant, the serum VD level remained in the normal range, the expressions of inflammatory cytokines were significantly reduced and eGFR was significantly recovered simultaneously in the treated group, while the VD insufficiency, the high expression of inflammatory cytokines and continuously decreased eGFR parallelly persisted in the untreated group. In this study, it was discovered for the first time that there is a negative correlation between vitamin D levels and the expression of multiple inflammatory cytokines in kidney transplant recipients (KTRs), while there is a significant positive correlation with renal function recovery. These findings suggest that vitamin D plays an important systemic anti-inflammatory role in maintaining immune homeostasis in KTRs. VD supplementation is beneficial to the control of systemic inflammation and immune activation in KTRs. The efficient restoration of VD/VDR interaction can suppress the expression of inflammatory cytokines, regulate immune-inflammatory responses, and prevent inflammation-related tissue damage, thereby preserving and safeguarding renal transplant function.

The inhibitory effect of VD/VDR on NF-κB-mediated inflammation has been demonstrated. Active binding of vitamin D to its receptor (VDR) exerts anti-inflammatory effects by inhibiting the activity of IκBα kinase (IKK), suppressing the expression of NF-κB inhibitory protein (IκBα), and reducing IκBα ubiquitination, thereby down-regulating NF-κB activity (32–34). VD/VDR signaling down-regulated the expression of pyroptosis associated markers NLRP3, GSDMD-N, Cleaved Caspase-1 and mature IL-1βin AKI models, and partially alleviated cisplatin induced acute kidney injury by inhibiting pyroptosis mediated by NF-κB (29). Kong F et al. (22) reported that genes overexpressed in T cells and B cells of CAMR KTRs were mainly enriched in inflammatory pathways, and NF-kB and MAPK signaling pathways were involved in inflammatory response, T cell activation and antibody-mediated rejection, mediating the occurrence and development of CAMR. Transplant rejection is the result of multiple factors. Combining the findings of this study, we concluded that vitamin D deficiency in kidney transplant recipients leads to a lack or insufficiency of vitamin D-mediated anti-inflammatory effects, rendering them unable to combat active inflammation and graft inflammatory injury caused by antigen stimulation, shifts in cellular homeostasis, and other factors. In the treatment group, KTRs with similar backgrounds of transplant rejection risk and immunosuppressive therapy were administered effective VD supplementation to correct their deficiency state. This resulted in the restoration of VD’s anti-inflammatory immunomodulatory effect. By inhibiting the NF-kB signaling pathway, which plays a crucial role in chronic rejection, the expression of related inflammatory cytokines is suppressed. This leads to a reduction in inflammation-mediated injury of the transplanted kidney and ultimately protects and maintains its function. The data from this study indicate a significant reduction in multiple cell-derived inflammatory cytokines among kidney transplant recipients who received effective VD treatment. Given that the vitamin D receptor (VDR) is expressed in various immune cells, it suggests that VD may have anti-inflammatory effects on multiple cellular targets in KTRs. However, further research is needed to identify specific cell targets and regulatory effects.

Although numerous studies suggest an association between vitamin D deficiency and poor prognosis in kidney transplantation (35), there is a lack of reliable data on the effectiveness of vitamin D supplementation in protecting renal function post-transplantation and improving long-term outcomes. Furthermore, the mechanism underlying its potential benefits remains unclear. This study focuses on the anti-inflammatory effect of vitamin D (VD) supplementation in kidney transplant recipients (KTRs) and its role in the graft function improvement. With the enrollment of one retrospective cohort and one prospective cohort of KTRs, vitamin D metabolism, inflammatory state, and renal graft function were tested, dynamically tracked and synthetically analyzed. To the best of our knowledge, this is the first study to investigate the therapeutic effect of vitamin D supplementation in kidney transplant recipients which paying close attention to the anti-inflammatory and immunomodulatory effects. Our findings confirm that vitamin D supplementation is beneficial for protecting and maintaining graft function in KTRs. Monitoring serum levels of inflammatory cytokines could serve as a promising biomarker for assessing the efficacy of vitamin D supplementation in kidney transplant recipients.

This study has some limitations. Firstly, the study had a relatively small sample size of kidney transplant recipients. Obtaining samples of kidney transplant recipients with biopsy results is difficult. Additionally, due to the extensive testing involved and high demand for sample volume per patient, only a limited number of eligible kidney transplant recipients were included in this clinical study cohort. In future follow-up studies, we aim to expand our sample size and carry out more in-depth and comprehensive research to increase the persuasiveness of the conclusion. Secondly, our monitoring of inflammatory cytokines was limited to samples taken at the initiation of VD supplementation and 12 months post-transplant. A more comprehensive study with a larger sample size and frequent monitoring would provide a more accurate depiction of the time course for the anti-inflammatory effects of VD supplement therapy, thus optimizing treatment strategies for kidney transplant recipients. In this study, we have only presented the data from an 18-month follow-up. A prospective study with a longer follow-up period is necessary to assess the impact of VD supplementation on long-term survival and maintenance of renal function in KTRs.

In summary, our findings suggest that sustained inflammatory activity and tissue damage mediated by high levels of inflammatory cytokines are important immune factors involved in chronic graft loss following kidney transplantation. Vitamin D deficiency in kidney transplant recipients (KTRs) impedes the proper anti-inflammatory function of vitamin D. Effective vitamin D supplementation contributes to controlling excessive immune inflammation and restoring immune homeostasis in KTRs. Early effective vitamin D supplementation after transplantation is beneficial.

## Data availability statement

The original contributions presented in the study are included in the article/supplementary material. Further inquiries can be directed to the corresponding author.

## Ethics statement

The studies involving human participants were reviewed and approved by the Ethics Committee of West China Hospital (Chi CTR2200056077). The patients/participants provided their written informed consent to participate in this study.

## Author contributions

Y-JB: Participated in research design, the writing of the paper, the performance of the research, and data analysis. Y-ML: Participated in the writing of the paper, the performance of the research, and data analysis. S-MH: Participated in the performance of the research and data analysis. Y-GZ: Participated in the performance of the research. Y-FA: Participated in the performance of the research. L-LW: Participated in research design. Y-YS: Participated in research design and the writing of the paper. All authors contributed to the article and approved the submitted version.
